# The impact of podoconiosis on quality of life in Northern Ethiopia

**DOI:** 10.1186/1477-7525-11-122

**Published:** 2013-07-17

**Authors:** Elizabeth Mousley, Kebede Deribe, Abreham Tamiru, Gail Davey

**Affiliations:** 1 Brighton and Sussex Medical School, Falmer, Brighton, United Kingdom; 2 School of Public Health, Addis Ababa University, Addis Ababa, Ethiopia; 3 International Orthodox Christian Charities, Debre Markos, Ethiopia

**Keywords:** Podoconiosis, Elephantiasis, Quality of life, Neglected tropical diseases, Ethiopia

## Abstract

**Background:**

Podoconiosis is one of the most neglected tropical diseases, which untreated, causes considerable physical disability and stigma for affected individuals. Little is known about the quality of life (QoL) of patients with podoconiosis. This study aimed to assess the QoL of patients with podoconiosis in comparison with healthy controls in Ethiopia.

**Methods:**

A comparative cross-sectional study was conducted in May 2012, among 346 clinically confirmed adult patients with podoconiosis, and 349 healthy adult neighbourhood controls in Dembecha woreda (district) in northern Ethiopia. QoL was assessed using the validated Amharic version of the World Health Organisation Quality of Life questionnaire (WHOQoL-BREF) scale; in addition, mental health and stigma were assessed by the Kessler-10 scale and podoconiosis stigma scale respectively. Logistic regression analysis was done to identify factors associated with QoL.

**Results:**

Patients with podoconiosis had significantly lower mean overall QoL than the controls (52.05 versus 64.39), and this was also true in all four sub domains (physical, psychological, social and environmental). Controls were 7 times more likely to have high (above median) QoL (Odds Ratio = 6.74, 95% Confidence Interval 4.62 to 9.84) than cases. Factors associated with lower QoL were: experiencing high levels of stigma, living in an urban area, being illiterate, having additional co-morbidities, and being unmarried. Mental illness was associated with lower scores in psychological and physical domains.

**Conclusions:**

Programs targeting podoconiosis interventions should include QoL as an indicator for monitoring progress. Interventions targeting improvement of QoL among patients with podoconiosis should address depression, stigma and other co-morbidities.

## Background

Podoconiosis is a form of elephantiasis that predominantly affects barefoot subsistence farmers in areas with red volcanic soil [1]. It has been identified as an important neglected tropical disease (NTD) in East Africa, South America and Asia [2-7], and is estimated to affect 1 million people in Ethiopia alone [8]. This neglected tropical disease is characterised by bilateral swelling of the lower legs with mossy and nodular changes to the skin, and causes considerable disability [1]. The aetiology is not fully understood, however current evidence suggests that mineral particles from irritant volcanic soils have a role, with some families having an additional genetic susceptibility to the condition [9,10]. Podoconiosis follows a chronic course, with progressively increasing disability, especially with continued exposure to irritant soils. However, with simple treatment, the condition is reversible.

The disability and deformity caused by podoconiosis have been shown to have economic and social consequences [11,12]. These include isolation, and exclusion from community events [13], but also difficulties finding employment, gaining education and getting married [12]. These are vital activities for both social and economic well-being. The economic costs of the condition are also high, with direct treatment costs being equivalent to US$143 per patient per year [11]. In addition, the physical challenges of the condition contribute to large productivity losses [11]. As the majority of patients are of working age, this has consequences for the wider community [3]. Podoconiosis has been found to be poorly understood by community members and health care professionals alike, with misconceptions about aetiology, infectiveness, and prognosis amongst others [12,14]. Associated with this, it has been shown that podoconiosis attracts a great deal of stigma, with many negative attitudes and beliefs found about podoconiosis in affected communities, as well as among patients themselves [7,14].

WHO defines Quality of Life as “individuals’ perception of their position in life in the context of the culture and value systems in which they live and in relation to their goals, expectations, standards and concerns” [15]. This is affected by “physical health, psychological state, level of independence, social relationships, personal beliefs and their relationship to salient features of their environment” [15]. Like most other categories of disease, NTDs including lymphatic filariasis (LF), leprosy, trachoma and Buruli ulcer [16] have been found to decrease quality of life. Neglected tropical diseases affect quality of life both physically, through pain, disability, and other symptoms such as itching, and socially, by reducing productivity, marginalisation and causing stigma [16]. The stigma associated with these conditions as been shown in turn to cause social isolation and exclusion, relationship difficulties including reduced marriage prospects, and negative feelings such as shame and lack of self-esteem [16]. These factors are also present in podoconiosis, which results in significant stigma, social costs and physical difficulties [11,12,14,17].

In a literature search, thirteen studies were found that assessed quality of life of patients with NTDs using standardised scales. One of these looked at dermatological quality of life in podoconiosis, and found a significant reduction in quality of life comparable to other dermatological conditions [18]; of the rest, five focused on leprosy [19-23], five on lymphatic filariasis or lymphoedema in general [24-28], and one each on trachoma [29] and cutaneous Leishmaniasis [30]. In addition, there was one qualitative study of lymphatic filariasis [31] which focused specifically on hydrocele in this group. This focus group and interview-based study found that LF hydrocele reduced physical activity and created work, marital and sexual problems, and stigma.

Overall, the review suggested that these NTDs have a significant impact on quality of life, no matter which of the assessment scales were used. However, few of the studies actually compared a patient group with healthy controls, so other factors might reduce the score for a community as a whole; having said this, those that did include controls tended to find worse quality of life in patients. Factors such as disability, deformity and discrimination, which have been found to increase the association between other NTDs and quality of life, are also found in podoconiosis. In addition, these studies consistently showed NTDs to exert the greatest effects in the physical and psychological domains.

This review leads to the hypothesis that podoconiosis, in common with the other NTDs, will have a significant impact on the quality of life of sufferers. Whilst there has been one study of the impact of podoconiosis on dermatological quality of life [18], there is a lack of literature on the more general effect of podoconiosis on quality of life. It is likely that podoconiosis has a large effect on quality of life of sufferers, and understanding this better is essential for establishing the full burden of the disease, planning appropriate services, and ensuring podoconiosis is treated as a high priority condition in local contexts.

## Methodology

### Study setting

The study took place in Dembecha woreda (district), part of the West Gojjam zone of Amhara regional state, northern Ethiopia, which has a population of 142,118. The woreda is split into 29 kebeles (the smallest administrative unit), of which 25 are rural and 4 are urban. Just 14% of the population live in urban areas. The largest ethnic group reported in Dembecha is Amhara (99.82%), and Amharic is spoken as a first language by 99.87%. The majority of the inhabitants of this region are Ethiopian Orthodox Christians (98.47%), while 1.46% are Muslim [32].

Podoconiosis has been found to be prevalent in this area. In November 2011, a baseline survey prior to establishing a podoconiosis treatment service in the area screened 51,017 individuals and identified 1,704 new cases of podoconiosis [33]. Between June 2010 and late 2011, the only podoconiosis services available near Dembecha were 45km away in the town of Debre Markos. Previous to this there were no podoconiosis treatment services in the entire Region. In 2011, the Debre Markos podoconiosis project expanded to 6 satellite sites, including Dembecha. The satellite sites offer 3 months of treatment (hygiene, secondary infections, wound care, bandaging, minor surgery and manual lymphatic drainage therapy), training in self care, and free shoes.

**Figure 1:**
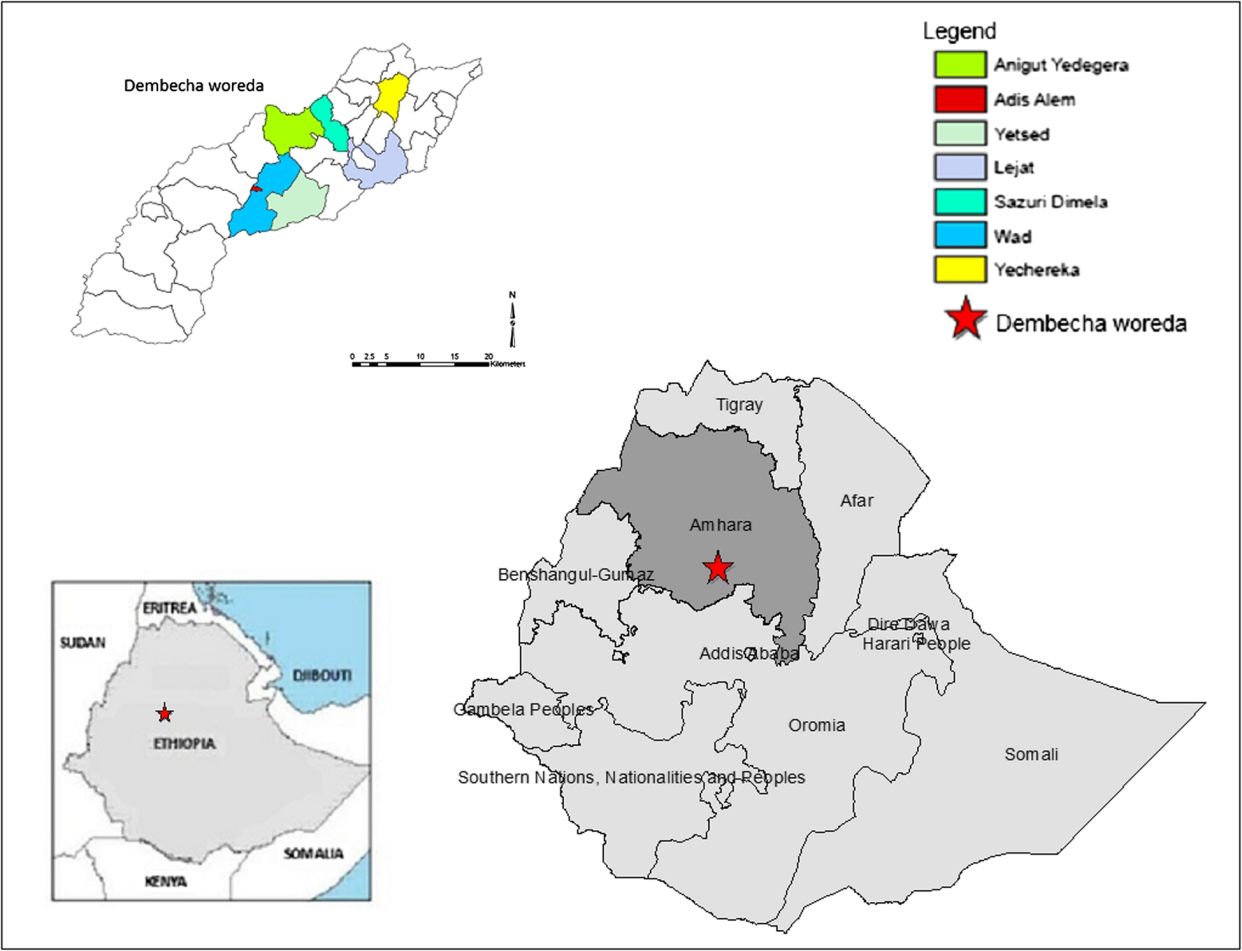
Map of the study area.

Seven kebeles within Dembecha woreda were selected for the study. These were chosen on the basis of having the highest prevalence of podoconiosis identified by the above survey, and so the largest possible patient population to sample. The study population constitutes those patients identified by the 2011 survey, and their neighbours without podoconiosis (Figure 1).

### Study design and participants

The study used a questionnaire-based, comparative cross-sectional study design. It compared two distinct study groups - a case group with clinically confirmed adult patients with podoconiosis, and healthy adult neighbours as controls. A quantitative methodology was chosen for this study, as the primary aim was to establish whether decreased quality of life was associated with podoconiosis.

Since the study was nested within one investigating mental health, the sample size was calculated to investigate differences in common mental disorder prevalence between podoconiosis cases and healthy controls. Epi-Info version 7.0 was used to calculate the number of cases and controls necessary to have 80% power to detect a 10% difference between the two groups with 95% confidence interval, based upon a 22.7% prevalence of mental disorder in the general population, as found in a previous study in Ethiopia [34]. It was calculated that 315 participants were needed in each group. The target sample size was 694 to take into account a predicted 10% non-response rate. The final sample had 695 participants, 346 of whom were podoconiosis cases, and 349, controls.

The cases were selected based on the population of patients with podoconiosis identified in the aforementioned survey. 50 patients from each kebele were selected. This was a near-total sample. Random selection was not practically possible due to the wide distribution of inhabitants within each kebele, and so convenience sampling was used, with the first 50 patients on each list who could be found being selected. The controls were non-affected individuals neighbouring the cases. It was decided, by picking a direction from a hat, that these would be from the house to the right of the case’s house to attempt to minimise bias due to discriminative selection by the data collectors. An attempt was made to match controls to cases based on age and gender, as these have been consistently shown to be strongly associated with levels of quality of life [35,36]. However, some of the data collectors found this was impossible in certain cases, leading to the matching protocol being inconsistently implemented. Participants under 15 years of age were excluded.

### Measurements

The primary variable of interest was quality of life, as measured by the WHOQoL-BREF [36]. Potential confounders included age, gender, education, marital status, place of residence, income, comorbidities, alcohol and substance use, stigma, common mental disorders, and podoconiosis stage. These were selected as they had previously been found to be factors associated with differences in quality of life in previous studies [37,38].

Three standardised and previously validated tools were used. The WHOQoL-BREF [36] was used to assess quality of life in all participants, the Kessler-10 scale [39,40] was used to assess mental health in all participants, and the podoconiosis stigma scale [41] was used to assess felt and enacted stigma among patients with podoconiosis only. In addition, demographic and social variables were assessed using a questionnaire adapted from previous studies, and piloted before the study.

The WHOQoL-BREF scale [36] assesses quality of life in 4 domains; physical health (7 items), psychological health (6 items), social relationships (3 items), and environmental health (8 items). In addition, 2 items assess general quality of life. This scale was developed by the WHO as a shortened version of the QOL-100. WHOQoL-BREF uses a 5-point scale for each answer, and these are scored positively, with higher values meaning a higher quality of life. Some negatively framed questions must be inverted before analysis. The domain scores are totalled and then converted to a 1–100 scale. The overall quality of life score is calculated as a simple addition of the scores for each question, and is on a 1 to 100 scale. The Amharic translation used has been previously validated and used in Ethiopia mainly in relation to post-conflict displacement [37,38,42,43].

The Kessler-10 scale [39,40] is a screening tool which measures the likelihood of some form of common mental disorder, such as depression or anxiety. The Amharic translation has previously been validated and used in Ethiopia for assessing common mental disorder in comparison with the gold standard of psychiatric assessment [42-44]. The Amharic translation found the optimum cut-off to be 16/17 for common mental disorders, with sensitivity of 84.2% and specificity of 77.8% [44]. Previous studies have found excellent reliability of the scale, with a reliability coefficient (Cronbach’s alpha) of between 0.90 and 0.93 [34,42-45].

The podoconiosis stigma scale [41] was developed specifically for patients with podoconiosis in Ethiopia, and has been previously validated [41]. It has 4 independent sections which assess felt and enacted stigma for patients and communities, and which are designed to be used together or separately. Only the patient scales were used for this study. The piloting of this scale found it had excellent reliability, with a reliability coefficient (Cronbach’s alpha) of 0.955 for patient felt stigma, and of 0.937 for patient enacted stigma [41].

### Data collection procedures

The questionnaires were piloted before the study. The pilot included seven patients and four controls who lived within the nearest large urban area, Debre Markos. Minor corrections were made to the wording of questions where these were unclear and to the layout to improve clarity during completion by the collectors.

Data collection was carried over 5 days between 3^rd^ and 7^th^ May 2012. The data were collected by 7 local nurses, 1 per kebele, who were familiar with the local area, had relevant medical background knowledge, and were fluent in Amharic, the participants’ main language. The questionnaires were used as the basis of structured interviews, rather than self-completed, due to low literacy levels. The data collectors received one day of training. This covered: the basics of podoconiosis and the staging techniques; the objectives of the study and its possible impacts; details of the questionnaire; taking informed consent; matching criteria and sampling techniques; and an opportunity to practice administering the question followed by a debrief. Three of the authors were supervisors (EM, KD, AT) and oversaw the data collection process; two of these (KD, AT) were fluent in Amharic. Each day the supervisors had contact with all the data collectors to answer any queries, and reviewed all of the questionnaires for missing data and inconsistencies. Questionnaires were returned for completion or correction if there were problems. In addition, supervisors checked the reliability of the data collectors by re-interviewing a number of participants. This found that the answers were broadly the same, and reliability of the data collection was not considered to be a problem.

### Data analysis

Data analysis was done using SPSS-19. Data were first checked and cleaned, with missing values on the scales replaced with the average of that item. Five questionnaires were excluded because they were incomplete.

Descriptive analysis of the social demographic distributions and the degree of comparability of the case and control groups was done. When comparing simple frequencies, the chi^2^ test was used to establish significance; when comparing means, the independent t test was used to compare the mean differences in QoL level across groups. Mean scores of the overall QoL, and 4 sub-domains, were compared. Presence of mental disorder was based on the cut-offs outlined above.

Logistic regression was performed for total QoL, and for each of the physical and psychological sub-domains. For the quality of life scores, the median of each scale was calculated and used as a cut-off point between “High QoL score” and “Low QoL score”. The medians were 58.5, 57.2, and 58.3 for total QoL, physical and psychological sub-domains respectively. For each dependent variable, all the important variables were tested, and those with a significant association were included in multivariate analysis, in addition to variables shown previously to impact upon the dependent variable. This meant that occupation was excluded from all as being not significant, and living in either rural or urban areas was excluded from physical domain analyses. In addition, stigma was excluded from the multivariate analysis as it was only measured for cases and not controls.

The reliability coefficients of the three scales and the correlation between the domains of the WHOQoL-BREF were also assessed.

### Regulatory approval and ethical issues

Ethical approval was sought and obtained from the Research Governance and Ethics Committee of Brighton and Sussex Medical School, and the Amhara Regional Health Bureau. Written informed consent was sought from all participants, with those who were unable to sign leaving a thumbprint instead. When individuals aged <18 years were encountered, consent was obtained from their parents or guardians. Data collectors were trained in key ethical issues, including how to ensure informed consent, as part of their initial briefing. All questionnaires were anonymised and identifiable information stored separately from the research data. Electronic data was kept on a password-protected computer.

## Results

### Demographic characteristics

The groups were well matched on place of residence and were comparable in terms of gender and age (Table 1). All those in the study were Amhara in ethnic origin. However there were some significant differences between the podoconiosis and healthy control groups. The podoconiosis group contained significantly more illiterate individuals (p=0.013) and a higher proportion of people with lower levels of education (p=0.006), particularly secondary education; lower average income (p<0.001) and more individuals in the lower income quintiles (p<0.001); and fewer married individuals (p<0.001). The distribution of occupations within the two groups was also significantly different, with the podoconiosis group containing fewer farmers and more traders than the healthy group.

**Table 1:** **Basic characteristics of the study groups**, **patients with podoconiosis and healthy controls**, **Northern Ethiopia**, **2012**

**Variables**		**Podoconiosis cases (%)**	**Healthy controls (%)**	**p value**^**1**^
Gender	Male	139 (40.2)	149 (42.7)	0.550^2^
	Female	207 (59.8)	200 (57.3)	
Age (years)	Mean (Standard Deviation)	45.74 (13.190)	44.63 (12.903)	0.272
	Range	15-80	15-85	
	15-24	10 (2.9)	16 (4.6)	0.495
	25-34	63 (18.2)	58 (16.6)	
	35-44	79 (22.8)	95 (27.2)	
	45-54	92 (26.6)	77 (22.1)	
	55-64	75 (21.7)	76 (21.8)	
	65+	27 (7.8)	27 (7.7)	
Village	Yechereka	50 (14.5)	50 (14.3)	1.000
	Adis Alem	55 (15.9)	57 (16.3)	
	Lejat	43 (12.4)	43 (12.3)	
	Sazuri Dimela	48 (13.9)	49 (14.0)	
	Wad	50 (14.5)	51 (14.6)	
	Yetsed	50 (14.5)	48 (13.8)	
	Anigut Yedegera	50 (14.5)	51 (14.6)	
Area of Residence	Rural	339 (98.0)	339 (97.1)	0.636
	Urban	7 (2.0)	10 (2.9)	
Occupation	Farmer	252 (72.8)	266 (76.2)	0.027
	Traders	36 (10.4)	30 (8.6)	
	Employed	6 (1.7)	7 (2.0)	
	Daily Labourer	21 (6.1)	14 (4.0)	
	Housewife	0	11 (3.2)	
	Student	4 (1.2)	4 (1.1)	
	Jobless	16 (4.6)	11 (3.2)	
	Other	11 (3.2)	6 (1.7)	
Ethnicity	Amhara	346 (100.0)	349 (100.0)	-
Literacy	Literate	81 (23.4)	112 (32.1)	0.013
	Not literate	265 (76.6)	237 (67.9)	
School Attendance (of literate)	No formal school	46 (56.8)	48 (42.9)	0.006
	Primary (grade 1–6)	27 (33.3)	41 (36.6)	
	Secondary (grade 7–12)	8 (9.9)	23 (20.5)	
Household Income (Birr/month)	Mean (SD)	285.14 (206.42)	384.3 (263.38)	<0.001
	Min-Max	0-1500	0-1500	
	1^st^ quintile (poorest)	86 (24.9)	53 (15.2)	<0.001
	2^nd^ quintile	77 (22.3)	69 (19.8)	
	3^rd^ quintile	79 (22.8)	54 (15.5)	
	4^th^ quintile	56 (16.2)	82 (23.5)	
	5^th^ quintile (richest)	48 (13.9)	91 (26.1)	
Marital Status	Married	198 (57.2)	265 (75.9)	<0.001*
	Unmarried	30 (8.7)	31 (8.9)	
	Divorced	63 (18.2)	34 (9.7)	
	Widowed	55 (15.9)	19 (5.4)	

1P value was calculated using Chi-square test, 2P value was calculated using t test to compare mean difference.

### Quality of life

Patients with podoconiosis had significantly lower mean overall QoL scores than the controls (Table 2). The overall mean QoL (SD) score among patients was 52.05 (10.71) and 64.39 (9.65) among healthy controls (p<0.001), with a mean difference of −12.35 (95% CI: -13.87 to −10.83). This was also seen in all four sub domains (physical, psychological, social and environmental) (p<0.001). The largest difference was seen in the physical domain, where the mean difference was −20.23 (95% CI: -22.58 to −17.87).

**Table 2:** **Quality of life scores for patients with podoconiosis and healthy controls**, **Northern Ethiopia**, **2012**

**Variables**	**Podoconiosis cases**	**Healthy controls**	**Mean difference (95% CI)§**	**p value**^**1**^
Overall WHOQoL-BREF Score Mean (SD)	52.05 (10.71)	64.39 (9.65)	−12.35 (−13.87 to −10.83)	<0.001
Physical Domain/100 Mean (SD)	47.89 (16.68)	68.12 (14.87)	−20.23 (−22.58 to −17.87)	<0.001
Psychological Domain/100 Mean (SD)	51.12 (18.18)	66.98 (17.38)	−15.85 (−18.51 to −13.21)	<0.001
Social Domain/100 Mean (SD)	52.12 (20.73)	67.16 (17.80)	−15.05 (−17.92 to −12.17)	<0.001
Environmental Domain/100 Mean (SD)	43.06 (17.31)	56.44 (15.57)	−13.37 (−15.83 to −10.92)	<0.001

§ Calculated using a two-sample t-test showing the mean difference.

### Factors influencing quality of life

Factors significantly associated with quality of life are depicted in Table 3. Healthy controls were 6.7 times more likely to have ‘high’ (above median) QoL than patients with podoconiosis (OR=6.74, 95% CI: 4.62 to 9.84). Other factors associated with below-average QoL scores included: experiencing high levels of stigma (OR=3.705, 95% CI: 2.19 to 6.27), living in an urban area (OR=0.19, 95% CI: 0.05 to 0.71), being illiterate (OR=2.07, 95% CI: 1.36 to 3.21), having additional co-morbidities (OR=2.12, 95% CI: 1.19 to 4.06), and being unmarried. The same factors affected the physical and psychological domains as the overall quality of life score. In addition, absence of mental disorder was associated with higher scores in the psychological (OR=1.98, 95% CI: 1.30 to 3.02) and physical domains (OR=1.81, 95% CI: 1.16 to 2.81), but not the overall QoL score (OR=1.21, 95% CI: 0.77 to 1.89).

**Table 3:** **Logistic regression for high quality of life scores**, **using a cut off of 58**.**50** (**the median**) **to divide into high and low quality of life scores on the WHOQOL**-**BREF**

**Variable**	**Categories**	**Low QoL score frequency (%)**	**High QoL score frequency (%)**	**Unadjusted OR (95% CI)**	**Adjusted OR§ (95% CI)**	**p value**
**Disease Status**	Podoconiosis Case	252 (72.8)	94 (27.2)	1	1	<0.001
	Healthy Control	96 (27.5)	253 (72.5)	7.06(5.06-9.86)	6.74 (4.62-9.84)	
**Gender**	Male	116 (40.3)	172 (59.7)	1.97 (1.45-2.67)	1.14 (0.74-1.75)	0.551
	Female	232 (57.0)	175 (43.0)	1	1	
**Income**	1^st^ quintile (poorest)	98 (70.5)	41 (29.5)	0.27 (0.16-0.44)	0.81 (0.42-1.55)	0.528
	2^nd^ quintile	76 (52.1)	70 (47.9)	0.59 (0.37-0.94)	1.09 (0.60-1.95)	0.783
	3^rd^ quintile	66 (49.6)	67 (50.4)	0.64 (0.40-1.04)	0.90 (0.50-1.63)	0.735
	4^th^ quintile	54 (39.1)	84 (60.9)	0.98 (0.61-1.60)	0.89 (0.50-1.60)	0.713
	5^th^ quintile (richest)	54 (38.8)	85 (61.2)	1	1	
**Area of residence**	Rural	335 (49.4)	343 (50.6)	1	1	
	Urban	13 (76.5)	4 (23.5)	0.30 (0.09-0.93)	0.19 (0.05-0.71)	0.013
**Literacy**	Literate	66 (34.2)	127 (65.8)	2.47 (1.75-3.49)	2.07 (1.36-3.21)	0.001
	Illiterate	282 (56.2)	220 (43.8)	1	1	
**Age** (**years**)	18-24	10 (38.5)	16 (61.5)	2.95 (1.12-7.76)	2.18 (0.65-7.37)	0.209
	25-34	61 (50.4)	60 (49.6)	1.81 (0.93-3.52)	1.49 (0.66-3.38)	0.334
	35-44	84 (48.3)	90 (51.7)	1.97 (1.05-3.72)	1.46 (0.68-3.12)	0.334
	45-54	82 (48.5)	87 (51.5)	1.95 (1.04-3.69)	1.84 (0.86-3.94)	0.119
	55-64	76 (50.3)	75 (49.7)	1.82 (0.96-3.46)	1.89(0.88-4.06)	0.102
	65+	35 (64.8)	19 (35.2)	1	1	
**Other health problems**	Yes	72 (77.4)	21 (22.6)	1	1	0.012
	No	276 (45.8)	326 (54.2)	4.05 (2.43-6.76)	2.20 (1.19-4.06)	
**Marital status**	Married	175 (37.8)	288 (62.2)	1	1	
	Unmarried	39 (63.9)	22 (36.1)	0.34 (0.20-0.60)	0.34 (0.17-0.71)	0.004
	Divorced	75 (77.3)	22 (22.7)	0.18 (0.11-0.30)	0.26 (0.13-0.49)	<0.001
	Widowed	59 (79.7)	15 (20.3)	0.15 (0.09-0.28)	0.31 (0.16-0.64)	0.001
**Stigma score**	High stigma score	142 (84.5)	68 (38.2)	1		
	Low stigma score	110 (61.8)	26 (15.5)	3.71 (2.19-6.27)		
**Common Mental Disorder**	No Common Mental Disorder	221 (44.8)	272 (55.2)	2.08 (1.49-2.92)	1.21 (0.77-1.89)	0.412
	Common Mental Disorder present	127 (62.9)	75 (37.1)	1	1	

§ adjusted for variables in the table.

**Figure 2:**
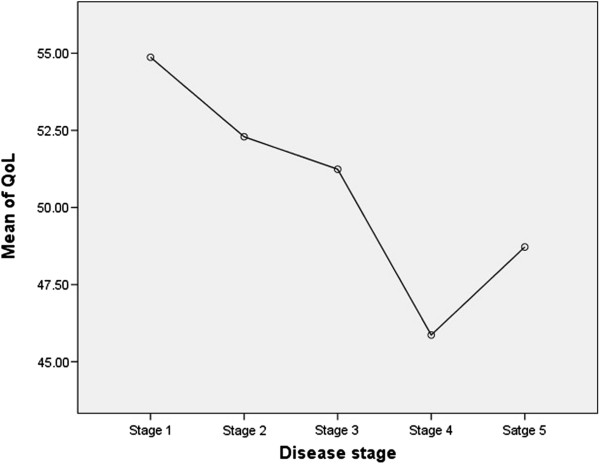
Mean quality of life by disease stage (significant at p=0.035).

Mean scores on the WHOQoL-BREF showed some association with disease stage, decreasing as the disease became more advanced; however the pattern was not consistent as stage 4 had lower mean QoL scores than stage 5 (Figure 2).

### Reliability of the tools used

All the tools were found to have good reliability, with Cronbach’s alpha of greater than 0.7. The Kessler, enacted stigma and overall QOL scales all showed excellent reliability, with a reliability coefficient exceeding 0.9 (Table 4). The four sub-domains of the WHOQOL-BREF were found to all have significant correlation, showing good reliability of this scale, and the domain scores (Table 5).

**Table 4:** Showing the reliability coefficient for the scales used within the study

**Scale**	**Reliability coefficient (Cronbach’s alpha)**
Kessler	0.940
Felt Stigma scale	0.842
Enacted Stigma scale	0.920
Total WHOQoL-BREF score	0.918
Physical Domain score	0.806
Psychological Domain score	0.795
Social Domain score	0.776
Environmental Domain score	0.725

**Table 5:** **Showing the correlation between the domains of the WHOQOL**-**BREF score for this study**

		**Physical QoL domain**	**Psychological QoL domain**	**Social QoL domain**	**Environmental QoL domain**
Spearman's rho(correlation coefficient)	Physical QoL domain	1			
	Psychological QoL domain	0.623*	1		
	Social QoL domain	0.669*	0.483*^*^	1	
	Environmental QoL domain	0.585*^*^	0.486*^*^	0.589*^*^	1

* Significant at p<0.01( two-tailed).

## Discussion

On the basis of evidence from other NTDs and the previous study of dermatological quality of life it was hypothesised that podoconiosis would have a large negative effect on quality of life. The results of this study support this hypothesis, with significant differences seen between patients and controls in mean scores of all of the domains of quality of life, and show an association between podoconiosis and poor quality of life. The regression analysis also supported this conclusion, and showed that patients with podoconiosis have almost seven times the risk of healthy people for having a below average score on this scale. This is in accordance with the only other study of quality of life in podoconiosis, which demonstrated low dermatological quality of life among patients with podoconiosis [18]. In addition, it is also consistent with what is already known about the social, psychological and economic effects of podoconiosis [11,12,14,17].

**Figure 3:**
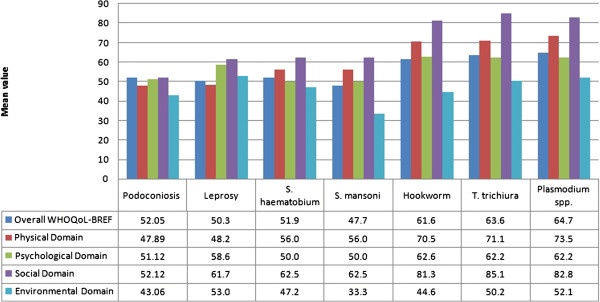
**Comparison of WHOQoL-BREF scores for different diseases [**[19]**,**[46]**].**

These results also fit well with what is known about other NTDs. When compared to the results of those studies that used the same scales, the impact of podoconiosis was found to be greater than other NTDs, particularly in the physical and social domains [23,28,46,47] (Figure 3, Table 6). This may be explained by the physical limitation caused by the leg swelling that characterises podoconiosis. In addition, previous studies have documented the social stigma due to podoconiosis [11,12,14,17]. Widespread misconceptions about podoconiosis may contribute towards stigma: studies have documented that most patients with podoconiosis and health providers believe that there is no treatment for podoconiosis [14,17]. For other diseases, particularly leprosy and lymphatic filariasis, patient are aware that treatment and cure exist. This, plus the low coverage of treatment services, may explain some of the differences found.

**Table 6:** Collated results of WHOQOL and DLQI questionnaire in use in neglected tropical diseases

**Study**	**Country**	**Disease**	**Sample**	Overall WHOQOL score (*denotes WHOQOL-BREF)
Joseph and Rao, 1999 [20]	India	Leprosy	50 treated patients 50 controls	94.5 101.5
Tsutumi et al., 2007 [23]	Bangladesh	Leprosy	Patients Controls	78.61 (male)*, 74.21 (female)* 86.64 (male)*, 91.09 (female)*
Costa et al., 2012 [19]	Brazil	Leprosy	120 patients	50.0*
Wejesinghe and Wickremasinghe, 2010 [28]	Sri Lanka	Lymphatic Filariasis	141 outpatients 128 healthy controls	70% score <12.5, 30% score >12.5† 24% score <12.5, 76% score >12.5†
Fürst et al., 2012 [46]	Côte d’Ivoire	S. haematobium	225	51.9*
		S. mansoni	225	47.7*
		Hookworm	225	61.6*
		T. trichiura	225	63.6*
		Plasmodium spp	225	64.7*
				**Overall DLQI score**
Chandrasena et al., 2007 [25]	Sri Lanka	Lymphatic filariasis	91 patients	8.2
Babu et al., 2006 [24]	India	Lymphatic Filariasis	203 patients	2.7
MacPherson, 2003 [26]	Guyana	Lymphatic Filariasis	15 patients	10.9
Legesse et al., 2008 [18]	Ethiopia	Podoconiosis	74 patients treated 74 patient untreated	3 13
Schuster et al., 2011 [47]	Brazil	Cutaneous Larva Migrans	91 patients	5
			11 Adults	6
			80 children	5

†raw scores were not reported in this study, instead those scoring above and below the median value were shown, Dermatological Life Quality Index(DLQI) values 0–1 no effect, 2–3 small effect, 4–8 moderate effect, 9–16 large effect, 17–24 very large effect [47].

**Figure 4:**
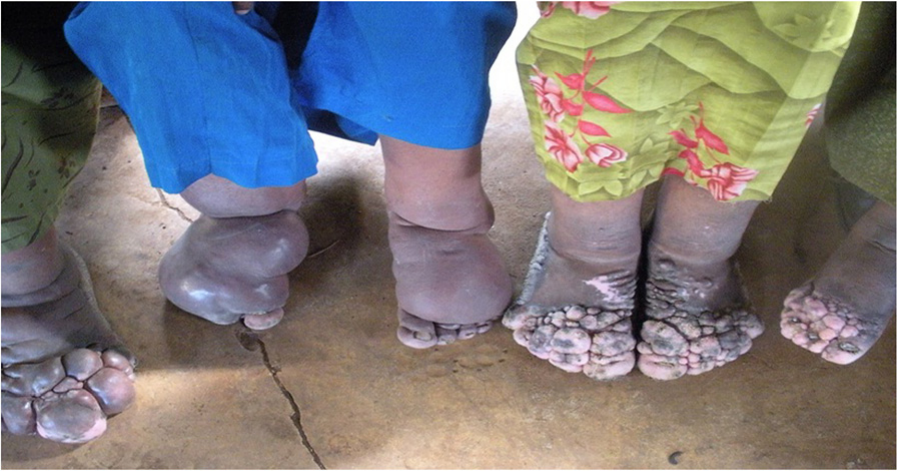
Picture of four female patients with podoconiosis from Ethiopia.

Most of the factors associated with QoL were consistent with previous reports. These included experiencing high levels of stigma, living in an urban area, being illiterate, having additional co-morbidities, and being unmarried. In addition, the presence of a mental health disorder was found to reduce physical and psychological quality of life, and this has been found to be a consistent predictor of low QoL in many studies [48,49]. Similarly, being unmarried has been found to be a predictor of low QoL [19]. It is also clear that other chronic health conditions are likely to reduce quality of life, for the same reasons as podoconiosis and the other NTDs. Stigma was also found to significantly reduce the QoL of patients with podoconiosis, as has been found for other NTDs [21,23] (Figure 4).

The study also found that the level of stigma experienced by patients increased and their QoL decreased as the disease stage became more severe. The most severe stages of podoconiosis are characterised by one or both of swelling above the knee and joint fixation in the feet, leading to greater physical disability and increased deformity. In addition to the physical limitations, these patients may experience more stigma and discrimination as they are easily identified. Similar findings have also been reported among patients with lymphatic filariasis and leprosy [21,25].

This study has several strengths. Firstly the study used validated instruments for measuring QoL, common mental disorders and stigma. Secondly the study included healthy controls against which to compare the QoL of patients with podoconiosis, which many of the previous studies in NTDs lack. Nonetheless the study is not without limitation. It could be argued that QoL is better measured qualitatively by exploring patients’ life experience and perceptions; however, as previous studies have documented the social exclusions and stigma faced by patients with podoconiosis, it was felt that the use of standardized tools to quantify the extent of QoL among patients with podoconiosis was a valuable addition to the evidence, and would enable direct comparison with other conditions. We did not measure the frequency of ‘acute attacks’ (variously termed acute adenolymphangitis or acute dermatolymphangioadenitis). Acute attack frequency appears to affect the QoL of patients with lymphatic filariasis, and might underlie some of the diminished life quality measured among patients with podoconiosis. Purposive sampling of the cases is also another limitation of the study. Nonetheless, more than 75% of the cases in the selected kebeles were included in the study.

Taking into account the above limitations, the results have a number of implications for podoconiosis prevention and treatment programmes. This study is the first to demonstrate the effect of podoconiosis on general quality of life, adding urgency to the task of reducing the burden of podoconiosis in affected communities. Previous studies have documented the effectiveness of even three months of simple hygiene and bandaging treatment for patients with podoconiosis, which leads to physical improvement of the feet and legs, with decreased swelling and mossy lesions [50,51]. This same treatment has been shown to be linked to significant improvement in DLQI among patients [18], suggesting that scale-up of this program would have considerable effects on quality of life of more patients. Addressing the stigma related to podoconiosis may also lead to improvement of QoL of patients. Media campaigns with clear messages that podoconiosis is preventable and treatable may counter widespread misconceptions and mitigate the associated stigma. Social support for patients by local associations may also mitigate internalized stigma and improve self-esteem.

In conclusion, this study has demonstrated that patients with podoconiosis have significantly lower QoL than healthy controls, both in the overall score and all domains of QoL. It has also shown that patients with podoconiosis have lower mean QoL scores than those with other NTDs such as lymphatic filariasis and leprosy. This indicates that programs focusing on treatment of patients with podoconiosis should follow a holistic approach to address the psychological, social and environmental impact of the disease, not just the physical illness. Efforts to improve QoL of patients should give priority to those with advanced disease, those who are unmarried, those with co-morbidities and those with low literacy.

## Competing interests

The authors declare that they have no competing interests.

## Authors’ contributions

EM, KD, GD conceived the study. EM, KD, AT conducted the field work. EM & KD conducted the analysis. EM, KD, AT and GD interpreted the findings. All authors read and approved the final manuscript.
